# Phenotypic characterization of presumptive propriospinal myoclonus in young dogs (2013-2025)

**DOI:** 10.1093/jvimsj/aalag211

**Published:** 2026-10-01

**Authors:** Dakir Polidoro, Sofie F M Bhatti, Patrick Santens, Paul Mandigers, Koen M Santifort, Shinichi Kanazono, Diane Lu, Daniel Sánchez-Masián, Alberta de Stefani, Intan Nur Fatiha Shafie, Patricia Álvarez, Nina Meyerhoff, Tadej Jeroncic, Serene R Lai, Guillaume F Dutil, Cecilia Rohdin, Adrien Marchand, Stephanie Thomovsky, Theofanis Liatis

**Affiliations:** AniCura Veterinary Practice Plantijn, 2600 Antwerp, Belgium; AniCura Vetimacs Medical Imaging, 1060 Brussels, Belgium; Faculty of Veterinary Medicine, Ghent University, 9820 Merelbeke-Melle, Belgium; Department of Neurology, Ghent University Hospital, 9000 Gent, Belgium; Evidensia Small Animal Referral Hospital Arnhem, 6825 MB Arnhem, The Netherlands; Department of Clinical Sciences, Utrecht University, 3584 CM Utrecht, The Netherlands; Evidensia Small Animal Referral Hospital Arnhem, 6825 MB Arnhem, The Netherlands; Evidensia Small Animal Referral Hospital Hart van Brabant, 5144 AM Waalwijk, The Netherlands; Veterinary Specialists and Emergency Center, Kawaguchi, Saitama 333-0823, Japan; Department of Veterinary Medicine and Surgery, University of Missouri, 65211 Columbia, United States; CityU Veterinary Medical Centre, Sham Shui Po, Hong Kong, China; Veterios Veterinary Hospital, 28022 Madrid, Spain; Royal Veterinary College, University of London, Hertfordshire, AL9 7TA, United Kingdom; Royal Veterinary College, University of London, Hertfordshire, AL9 7TA, United Kingdom; Department of Veterinary Clinical Studies, Faculty of Veterinary Medicine, University Putra Malaysia, 43400, UPM Serdang, Selangor, Malaysia; IVC Evidensia, Pride Veterinary Referrals, DE24 8HX Derby, United Kingdom; Department of Small Animal Medicine and Surgery, University of Veterinary Medicine Hannover Foundation, 30559 Hanover, Germany; Department of Small Animal Medicine and Surgery, University of Veterinary Medicine Hannover Foundation, 30559 Hanover, Germany; Maritime Veterinary Emergency and Specialty Centre, B3A 4Y8 Dartmouth, Canada; CHV Atlantia, 44200 Nantes, France; Department of Clinical Veterinary Medicine, VetSuisse Faculty, 3001 Bern, Switzerland; AniCura Djursjukhuset Albano, 182 36 Danderyd, Sweden; Centre Hospitalier Vétérinaire Saint-Martin, 74350 Allonzier-la-Caille, France; Department of Veterinary Clinical Sciences, Purdue University College of Veterinary Medicine, 47907 West Lafayette, United States; Plakentia Veterinary Clinic – Alimos, Athens, 174 55, Greece; School of Veterinary Medicine, European University Cyprus, Nicosia, 1516, Cyprus

**Keywords:** canine, extensor myoclonus, hyperkinetic, limb jerks, spinal movement disorder

## Abstract

**Background:**

Propriospinal myoclonus (PSM) is characterized by repetitive, brief jerks of the trunk, hips, and knees in humans and has not been reported in dogs.

**Hypothesis/Objectives:**

To describe phenotype, diagnostic findings, treatment, and outcomes in presumptive PSM in dogs.

**Animals:**

Twenty-seven dogs with presumptive PSM.

**Methods:**

Multicenter, retrospective study of dogs with myoclonus affecting the limbs and trunk without head involvement, identified through online calls for suspected cases.

**Results:**

Median age at onset was 9 months (range, 2-44 months). Propriospinal myoclonus involved pelvic limbs in all dogs, thoracic limbs in 14/27 (52%), and trunk in 9/27 (33%). Pelvic limb myoclonus was extensor in all dogs, whereas thoracic limb involvement was extensor in 12/27 (44%) and flexor in 3/27 (11%) dogs. Episodes were observed in a non-weight-bearing posture in all dogs, mainly lateral (25/27; 93%) and sternal (12/27; 44%) recumbency. Neurological examination was normal in all dogs. Abnormalities were not detected on magnetic resonance imaging of the spinal cord (4/6) or brain (6/6) in 5/6 dogs; in the remaining dog, findings were unrelated to the presumptive PSM. Follow-up information was available for 26/27 (96%) dogs. Sixteen dogs (59%) received no treatment, of which 9/16 (56%) showed spontaneous reduction in PSM frequency. Treatment with various antiseizure medications was started in 11/27 (41%) dogs, of which 5/11 (45%) improved.

**Conclusions and clinical importance:**

Presumptive PSM affected young dogs and is likely idiopathic. The condition is characterized by extensor myoclonus predominantly affecting pelvic limbs during recumbency, with preserved mentation. Spontaneous improvement seems common.

**Abstract Translated to Dutch/Flemish:**

Achtergrond: Propriospinale myoclonus (PSM) wordt bij mens gekenmerkt door repetitieve, schokbewegingen van de romp, heupen en knieën. Tot op heden is deze aandoening niet beschreven bij honden.

Doelstelling; Het beschrijven van het fenotype, diagnostische bevindingen, behandeling en klinische uitkomst.

Dieren: Zevenentwintig honden met vermoedelijke PSM. Methoden—Multicentrische retrospectieve studie bij honden met myoclonus waarbij de ledematen waren betrokken, geïdentificeerd via online meldingen van vermoedelijke gevallen.

Resultaten: De mediane leeftijd bij aanvang van de symptomen bedroeg 9 maanden (spreiding: 2-44 maanden). Bij alle honden was PSM aanwezig in de achterpoten, bij 14/27 (52%) in de voorpoten en bij 9/27 (33%) in de romp. De myoclonus van de achterpoten was bij alle honden in extensie. Ter hoogte van de voorpoten was de myoclonus in extensie (12/27 (44%) en in flexie (3/27 (11%). De episoden traden voornamelijk op in zijligging (25/27; 93%) en sternale (12/27; 44%). Neurologisch onderzoek was bij alle honden normaal. Magnetische resonantiebeeldvorming van ruggenmerg (4/6) en hersenen (6/6) was bij 5/6 honden normaal. Follow-upinformatie was beschikbaar voor 26/27 (96%) honden. Zestien honden (59%) kregen geen behandeling, waarvan 9/16 (56%) een spontane afname van de frequentie van de PSM vertoonden. Bij 11/27 (41%) honden werd een behandeling met aanvalswerende medicijnen gestart, waarbij 5/11 (45%) een verbetering vertoonden.

Conclusies en klinische relevantie: Vermoedelijke PSM lijkt voornamelijk voor te komen bij jonge honden en is waarschijnlijk idiopathisch. Het klinisch beeld wordt gekenmerkt door een myoclonus in extensie van voornamelijk de achterpoten tijdens het liggen. Een spontane verbetering lijkt vaak voor te komen.

## Introduction

Myoclonus is defined as a sequence of repeated, variably rhythmic, brief shock-like jerks, resulting from sudden involuntary contraction (positive) or relaxation (negative) of 1 or more muscles.^1^^,^^2^ In human medicine, different ways to classify myoclonus have been proposed.^3^ Recently, a new classification has been introduced, incorporating the clinical features, anatomical distribution, neurophysiological markers, and etiology of myoclonus. This classification is structured around 2 axes: Axis I, which provides a detailed clinical characterization of patients with myoclonus, and Axis II, which addresses etiology.^4^ The use of these classifications in veterinary medicine is challenging. For this reason, myoclonus is simply classified according to its topographic distribution (focal or generalized), association with epilepsy (epileptic or nonepileptic myoclonus), and myoclonic epileptic seizures.^1^^,^^2^

Propriospinal myoclonus (PSM) is a rare form of nonepileptic myoclonus in humans.^5–7^ It is a hyperkinetic movement disorder characterized by irregular flexor jerks involving the trunk and proximal limb muscles, particularly the hips and knees.^5^^,^^6^^,^^8–10^ These jerks are termed PSM as they are believed to originate from a spinal generator and are generally stimuli-induced, tending to increase in supine position (dorsal decubitus).^5^^,^^6^^,^^11^

The diagnosis of PSM is based on video-polymyography recordings of the myoclonic jerks, which present characteristic features, including discharges lasting from 50 to 300 ms and a slow spinal cord conduction velocity (5-15 m/s).^5^^,^^7^^,^^11–13^

In veterinary medicine, several etiologies of myoclonus are reported, such as degenerative encephalopathy-associated myoclonus (eg, Lafora disease, neuronal ceroid lipofuscinosis),^1^ myoclonic epileptic seizures (eg, juvenile myoclonic epilepsy associated with *DIRAS1* gene mutation, feline audiogenic reflex seizures and spontaneous late-onset myoclonic epilepsy in cats and dogs),^14–17^ drug-induced myoclonus (eg, opioids and propofol),^18–27^ encephalitis-associated myoclonus (eg, canine distemper virus infection),^28^^,^^29^ intoxication (eg, fluralaner, marijuana),^30^^,^^31^ and acute post-hypoxic myoclonus.^32^ Literature on the treatment of myoclonus in dogs and cats is scarce, but the use of levetiracetam seems to provide some benefit in cases of epileptic myoclonus.^1^^,^^17^^,^^33–36^

Although PSM has been described in humans, there are no published reports of this condition in dogs. The aim of this study was to describe a phenotypic homolog of PSM in dogs and report the diagnostic findings, treatment strategies and clinical outcome.

## Materials and methods

This multicenter, retrospective study included dogs presenting with episodes resembling human PSM. Ethical approval was not required since all data were retrieved retrospectively from clinical records and no experimental procedures were performed.

Cases were collected by actively searching for past posts using the keywords “limb myoclonus”, “limb spasms”, “limb jerks” and “kicking”, as well as through open calls in online veterinary forums (Veterinary Information Network-VIN® American College of Veterinary Internal Medicine-ACVIM/European College of Veterinary Neurology-ECVN Neurologists ListServe). Colleagues worldwide were invited to report potential cases. Inclusion criteria were (1) complete medical records, (2) dogs presenting with episodes of myoclonus of the limbs and trunk muscles without head involvement, (3) availability of video recordings of the episodes for review; and (4) examination by a board-certified neurologist or a neurology resident under the direct supervision of a board-certified neurologist (ECVN or ACVIM).

Clinical data was retrieved from medical records and included signalment, medical history, and clinical findings from physical and neurological examinations. Myoclonus semiology was evaluated based on video recordings and included distribution of affected body regions and symmetry, presence of flexion or extension movements, posture and mentation during episodes, behavioral responses, distractibility, and potential triggering factors. Diagnostic test results, when available, included complete blood count (CBC) and serum biochemistry profile, magnetic resonance imaging (MRI), computed tomography (CT), cerebrospinal fluid (CSF) analysis, and electromyography (EMG). Magnetic resonance imaging was performed using either high- or low-field magnets (Supplementary material S1). Treatment protocols and follow-up information were obtained from medical records and communication logs. Improvement was defined as complete resolution (ie, complete cessation of episodes) or a reduction in episode frequency or intensity, with or without treatment.

## Results

Twenty-seven dogs met the inclusion criteria over 12 years (2013-2025). Cases were collected from 17 veterinary institutions and private hospitals across the Netherlands (*n* = 5), United Kingdom (*n* = 4), Belgium (*n* = 3), France (*n* = 2), Germany (*n* = 2), Greece (*n* = 2), Hong Kong (*n* = 2), Japan (*n* = 2), Spain (*n* = 2), United States of America (*n* = 2) and Sweden (*n* = 1).

### Signalment and examination

Thirteen dogs (48%) were male (5 neutered), and 14 (52%) were female (5 neutered). Median age at onset was 9 months (range, 2-44 months). Breeds included crossbreed (4/27; 15%), Pomeranian (3/27; 11%), Miniature Poodle (3/27; 11%), Border Collie (2/27; 7%), Dachshund (2/27; 7%), Shih Tzu (2/27; 7%). The remaining breeds (1/27; 4% each) were Border Terrier, Chihuahua, Cocker Spaniel, French Bulldog, German Shepherd Dog, Hungarian Vizsla, Labradoodle, Miniature Pinscher, Miniature Schnauzer, Norfolk Terrier, and Yorkshire Terrier. Dietary Information at disease onset was available for 22 dogs (81%). Most dogs (17/22; 77%) were fed commercial diets, while 5/22 (23%) were receiving gluten-free diets. Neurological and general physical examination findings were normal in all dogs at presentation.

### Occurrence of episodes

Episodes occurred spontaneously during rest in all dogs. In 1 dog (1/27; 4%), episodes were also observed during gait. Triggers were identified in 4 dogs (15%), including exercise (*n* = 2) and stressful events (*n* = 1), although episodes also occurred in the absence of these triggers. Interestingly, in the remaining dog, episodes were consistently triggered when being lifted, with cessation upon returning to a weight-bearing posture or recumbency. Daily episodes were reported in 16/27 dogs (59%). The remaining dogs showed irregular patterns, with clusters of episodes followed by periods of resolution lasting days, weeks, or months.

### Characterization of the episodes

Episodes were bilaterally symmetric and occurred in a non-weight-bearing posture in all dogs, mainly in recumbency. The most common positions were lateral (25/27; 93%) and sternal (12/27; 44%) recumbency, followed by dorsal recumbency (4/27; 15%). Episodes were also observed in some dogs in upright stance (3/27; 11%) and sitting position (6/27; 22%; Video S1). Pelvic limbs were affected in all dogs, thoracic limbs in 14/27 (52%), and trunk muscles in 9/27 (33%). Extensor arrhythmic myoclonus was seen in the pelvic limbs in all dogs and in the thoracic limbs in 12/27 (44%), while a concomitant flexor myoclonus involving the thoracic limbs was seen in only 3/27 dogs (11%). Barking or growling behavior directed toward the pelvic limbs during the episodes was observed in 12/27 dogs (44%; Video S1). Episodes were distractible by tactile or auditory stimulation in 17/27 dogs (63%). Episode duration varied both between and within dogs. Most dogs (16/27; 59%) experienced episodes lasting less than 10 minutes, whereas 4/27 dogs (15%) had episodes lasting 1-2 hours. All dogs maintained normal mentation throughout episodes. No autonomic signs, post-ictal behavior, or delayed recovery were observed, as all dogs snapped out of episodes immediately.

### Diagnostic evaluation

Blood testing, including CBC and serum biochemistry profile, was performed in 23/27 dogs (85%). Abnormalities were detected in 2 dogs (9%). One dog had mildly non-specific low urea (2.4 mmol/L; reference value 2.5-9.6 mmol/L) and aspartate aminotransferase (AST) activity (65 U/L; reference value <50 U/L). The other dog had abnormally high serum bile acids (reference value <25 μmol/L); however, the clinical record reported the result only qualitatively as "high" without a numerical concentration. Despite normal ammonia levels (15 μmol/L; reference value <60 μmol/L), a liver disease was suspected, and the dog underwent abdominal CT, which did not reveal any abnormalities. In the absence of abnormalities on CT, portal vein hypoplasia was considered the most likely diagnosis. Liver biopsies and medical treatment for the suspected liver disease were recommended but declined by the owner, as myoclonus was the dog's only clinical complaint. Anti-gliadin antibody (IgG) concentration was measured in 1 dog and was increased (1.3 U/mL; reference value <0.6 U/mL). Canine distemper virus PCR and antibody titers were performed on whole blood in 1 dog, and both results were negative. One dog tested negative for the juvenile myoclonic epilepsy (*DIRAS1* gene mutation). Urinalysis was performed in 5/27 dogs (18%), and abnormalities were not detected in any case.

Magnetic resonance imaging was performed in 6/27 dogs (22%), including head and thoracolumbar vertebral column (1/6), head and cranial cervical vertebral column (1/6), head and whole vertebral column (2/6), and only head (2/6). Brain and spinal cord were normal in 5 dogs. One dog was diagnosed with Chiari-like malformation and mild cervical syringomyelia. One dog was submitted to a full-body CT scan and showed no abnormalities. Cerebellomedullary cistern CSF analysis was performed in 5/27 dogs (18%) and revealed no abnormalities. PCR testing for infectious diseases, including canine distemper virus, *Cryptococcus* spp., *Neospora caninum*, *Toxoplasma gondii*, *Ehrlichia canis*, and *Rickettsia rickettsia*, was performed on CSF in 2/27 dogs (7%), with negative results. Electromyography under sedation was performed in 1 dog, and no spontaneous electrical muscle activity was detected; however, no myoclonus was present during the study.

### Clinical course, treatment, and outcome

Sixteen dogs (59%) received no treatment, while 11/27 dogs (41%) did receive medical therapy. Follow-up information was available for 26/27 dogs (96%) with a median follow-up duration of 8 months (range, 1-136 months).

Among untreated dogs, episodes resolved spontaneously in 7/16 dogs (44%), decreased in frequency in 2/16 (12%), remained unchanged in 5/16 (31%), increased in frequency in 1/16 (6%), and 1/16 dog (6%) was lost to follow-up. Among treated dogs, 5/11 (45%) showed improvement. Two dogs achieved complete resolution: 1 treated with levetiracetam (20 mg/kg PO q8h) and 1 with fluoxetine (1 mg/kg PO q12h). Both remained episode-free at 18-month and 1-month follow-up, respectively, and continued to be episode-free after discontinuation of treatment, with levetiracetam stopped after 8 weeks and fluoxetine after 3 weeks. Three additional dogs showed partial improvement. One dog received, in different periods, several medication trials, including diazepam, trazodone, alprazolam, levetiracetam, topiramate, and fluoxetine. There was only improvement when topiramate (5 mg/kg PO q12h) was combined with fluoxetine (1 mg/kg PO q12h); after 4 months of treatment, episodes were still present, though at a lesser frequency. In another dog, there was only improvement when clonazepam (0.5 mg/kg PO q12h) was combined with levetiracetam (20 mg/kg PO q8h). But this same dog, in different periods of time, was previously trialed on other drugs such as diazepam, trazodone, alprazolam, and levetiracetam, without any improvement. Thirty-six months after the first episode, this dog was diagnosed with immune-mediated hemolytic anemia and died due to this condition. During that period, the dog was still having the episodes with the same frequency, but less intense. In the remaining dog, there was only improvement (decrease in frequency) when gabapentin (10 mg/kg PO q8h) was added to a previous non-effective phenobarbital trial (4 mg/kg PO q12h). Precise timelines between treatment initiation and clinical response were unavailable in most cases. Six treated dogs (54%) did not improve. Three received levetiracetam monotherapy (20-40 mg/kg PO q8h), with no benefit. One of these dogs was euthanized at the owner’s request due to lack of response to treatment, as it continued to experience daily, high-frequency episodes occurring in clusters. Despite being offered additional treatment options and alternatives, the owner elected to have euthanasia performed elsewhere. Within the other 2 dogs that have received only levetiracetam, the episode frequency remained the same in one, and increased in the other. The remaining 3 dogs received multiple medications including levetiracetam (3/3), clonazepam (1/3), phenobarbital (2/3), carbamazepine (2/3), zonisamide (2/3), fluoxetine (3/3), acetazolamide (1/3), and prednisolone (2/3), without clinical improvement.

## Discussion

This retrospective case series describes 27 young dogs with presumptive PSM, characterized predominantly by episodes of extensor myoclonus of the pelvic limbs occurring mainly in non-weight-bearing postures, with normal neurological examination between episodes in all dogs. While improvement was observed in 45% of dogs receiving medical treatment, spontaneous improvement occurred in 44% of untreated dogs. Although an experimental model of propriospinally-mediated myoclonus is reported in cats,^37^ to the authors’ knowledge, no previous reports have described myoclonus in dogs clinically resembling PSM in humans. The present study therefore provides a description of a phenotypic homolog of PSM in dogs, including clinical features, diagnostic findings, treatment, and outcomes.

Myoclonus of spinal cord origin can be subdivided into focal spinal myoclonus (formerly termed segmental), which does not propagate, and PSM, which spreads up and down the spinal cord rather than being limited to 1 or a few segmentally innervated muscles.^7^^,^^38^ In humans, focal spinal myoclonus is consistently associated with focal spinal cord lesions, either inflammatory, vascular, neoplastic, or traumatic.^12^^,^^39^ In dogs, cervical focal spinal myoclonus is reported associated with cervical intervertebral disc extrusion^40^ or less commonly with other cervical myelopathies.^41^ Spinal myoclonus occurs in association with the canine distemper encephalomyelitis.^42^ Kicking out of the pelvic limbs is described in young Cavalier King Charles Spaniels as part of a head/foot irritation syndrome; however, it was not directly proposed by the author as being potentially associated with PSM.^43^

All dogs in this study exhibited presumptive PSM episodes primarily in non-weight-bearing postures, particularly during recumbency, the vast majority in lateral recumbency and half of the cases in sternal recumbency. A smaller number of dogs presented the episodes while in dorsal recumbency. In 9 dogs, episodes were observed while in a weight-bearing posture, such as upright stance and sitting position. This finding parallels observations in humans, in whom symptoms are frequently triggered in the supine position and during relaxation or sleep onset.^6^^,^^38^ Position-dependent modulation of propriospinal excitability and reduced supraspinal inhibitory control during relaxation might contribute to this phenomenon.^38^^,^^44^ In dogs, the propriospinal tract (fasciculus proprius) facilitates integration of neural activity between spinal cord segments and between axial and limb muscles.^45^^,^^46^ Alterations in this system during non-weight-bearing postures might predispose to abnormal motor discharges. External sensory stimuli frequently trigger PSM in humans as well, such as external sensory stimulations of the abdominal wall, deep tendon reflex, or muscle stretch.^7^^,^^47^^,^^48^ In contrast, none of the dogs in this study exhibited stimulus sensitivity. Instead, episodes were often temporarily suppressed by tactile or auditory distraction.

Although episodes in dogs were most prominent during rest, owners did not report episodes during sleep. However, given the retrospective nature of the study, precise information regarding the timing of episodes in relation to sleep–wake transitions was not available. Based on video recordings, many dogs appeared distressed by the episodes and frequently changed position or stood up after a series of jerks (Video S1). Approximately half of the dogs directed attention, vocalization, or aggression toward the affected limbs (Video S1). In humans, episodes often cease during walking,^6^^,^^47^ whereas, in this study, a minority of dogs continued to exhibit episodes during stance or gait.

The main differential diagnosis in dogs with presumptive PSM episodes is epileptic seizures, particularly myoclonic epileptic seizures and clonic seizure activity. In human medicine, classification as a myoclonic epileptic seizure relies on the association between myoclonic jerks and concurrent changes on electroencephalography (EEG).^49^ In veterinary medicine, however, EEG is not widely available, despite its use in recent studies to characterize myoclonic epileptic seizures and epileptic myoclonus in dogs.^14^^,^^50^^,^^51^ Nevertheless, accurately characterizing the nature of myoclonus remains challenging without EEG confirmation, and interpretations should therefore be made with caution. However, myoclonic epileptic seizures are often described in patients in whom epilepsy represents the primary clinical disorder, with myoclonus occurring as an associated feature.^1^ None of the dogs in this study exhibited any other seizure type or other paroxysmal events aside from the presumptive PSM episodes. Furthermore, myoclonic epileptic seizures in juvenile myoclonic epilepsy, despite their strong association with the sleep–wake transition, and myoclonus in Lafora disease, for example, are often stimulus sensitive, with episodes triggered by visual or auditory stimuli. Myoclonic epileptic seizures in these conditions might progress to generalized tonic–clonic epileptic seizures.^1^^,^^2^ The absence of orofacial involvement, preservation of consciousness during episodes, lack of pre- and post-ictal phases, as well as autonomic signs, and poor response to antiseizure medications further support that presumptive PSM is most likely a nonepileptic movement disorder.

Propriospinal myoclonus is considered a painless condition in humans and is usually characterized by repetitive, generally flexor more than extensor, arrhythmic jerks, involving the trunk and proximal limb muscles, particularly the hips and knees.^6^^,^^12^^,^^38^ In contrast, the dogs in this study mainly exhibited extensor myoclonus of the pelvic limbs, with less frequent trunk muscle involvement. The predominance of extensor activity in dogs might reflect species-specific neuroanatomical and postural differences. In quadrupedal species, extensor muscles play a critical role in antigravity postural support and are subject to strong supraspinal inhibitory control.^45^ Disruption of this modulation might preferentially manifest as extensor hyperactivity. In contrast, in humans, flexor predominance is more common in upper motor neuron disorders.^52^^,^^53^ These anatomical and functional differences between quadrupedal and bipedal postures might help explain this variation in both species. On the other hand, as typically described in humans,^13^ the pattern of activation of the PSM in these dogs was predominantly bilateral and symmetric.

In this study, affected dogs represented multiple breeds, but crossbreed dogs were more frequently reported (4/27; 15%). There was no sex predisposition, and they were predominantly young, with a median age at onset of 9 months. In humans, PSM similarly shows no consistent sex predilection, although a slight male predominance has been reported, and typically affects adults with a mean age of onset of approximately 43 years.^6^ This contrasts with dogs, in which affected individuals are typically younger, reflecting a distinct difference in age of onset between the 2 species. Nevertheless, the occurrence in younger people has also been reported.^54^^,^^55^

In humans, most cases of PSM are considered idiopathic,^38^ although the existence of a truly idiopathic form remains controversial.^56^ Many cases previously classified as idiopathic might represent functional PSM due to lack of standardized diagnostic measurements and neurophysiological confirmation.^12^ Electrophysiological evaluation, including polymyography combined with jerk-locked back-averaging of EEG–EMG recordings, might assist in the assessment of suspected PSM by identifying a Bereitschaftspotential,^38^ also called “premotor potential”.^57^ This premotor potential, characterized by a slow negative cortical shift preceding voluntary movement, supports a functional origin when present.^10^^,^^58^ Accordingly, detection of a premotor potential is considered a useful marker in favor of a functional movement disorder and might help exclude a primary structural origin of the jerks. However, its diagnostic utility is limited, as the absence of a premotor potential does not exclude a functional etiology nor definitively rule out cortical involvement.^38^^,^^59^ Rarely, PSM has been associated with structural spinal cord lesions,^57^^,^^60–64^ but in most cases, no lesions were found, suggesting that the majority of patients whose clinical presentation resembles PSM are probably of psychogenic origin and not necessarily an organic disorder.^6^^,^^10^^,^^57^^,^^60^ This increases the suspicion that PSM is actually a functional movement disorder not associated with any structural neurological disease.^7^

A systematic review reported that 58% of PSM human cases were functional movement disorders, 16% idiopathic, and 26% symptomatic.^6^ The latter would include structural spinal cord lesions such as ischemic myelopathy, cervical neoplasia, syringomyelia, disc herniation, and trauma.^6^ Symptomatic PSM was also associated to other causes such as Lyme neuroborreliosis,^61^ gluten sensitivity,^62^ myasthenia gravis,^9^ paraneoplastic disease,^63^ and inflammatory processes.^44^ Less frequently, symptomatic PSM have also been drug-induced.^64–67^ Although several drugs used to treat PSM in these dogs, including gabapentin, carbamazepine, prednisolone, phenobarbital, and fluoxetine, have been reported to potentially induce myoclonus in humans,^68^ none of the dogs in this cohort had received these or any other drugs at the time of initial clinical onset. In the present study, the presumptive PSM was considered idiopathic, as no consistent neurological deficits or structural abnormalities were identified in most dogs. However, classifying a disease as functional is not yet a common practice in veterinary neurology. Notably, distractibility, vocalization, and spontaneous resolution are clinical hallmarks of functional movement disorders and more specifically of functional PSM in humans.^6^ In our study, distractibility, vocalization and spontaneous resolution were observed in 63%, 44%, and 26% of the dogs, respectively, suggesting that a functional component cannot be fully excluded.

Currently, in humans, there are no standardized treatment guidelines for PSM; instead, treatment is largely dependent on the underlying cause, if ever diagnosed.^38^ Idiopathic PSM is often refractory to pharmacological treatment.^13^ And symptomatic drug therapy in these cases is often disappointing.^12^ In a review, PSM resolved with treatment in only 23% of the cases, after predominantly pharmacological treatment as well as non-pharmacological approaches, including surgery, diet change and electrotherapy.^6^ Botulinum toxin and clonazepam are the main drugs reported in the treatment of PSM in humans, while the latter seemed to be effective in roughly 52% of the cases.^6^ Levetiracetam, when combined with clonazepam, is reported to have a synergistic effect in decreasing myoclonic activity.^69^ Serotonin reuptake inhibitors, such as fluoxetine, are reported to be effective in a few cases.^70^ In humans, although zonisamide is primarily used to treat cortical myoclonus,^71^ limited efficacy has been reported in PSM, but only in a small proportion of cases.^13^^,^^47^ Other drugs, such as carbamazepine, valproic acid, prednisone, and baclofen, are mostly reported as ineffective in the treatment of PSM.^6^ Dietary changes, such as implementation of a strict gluten-free diet, have also shown some promise in helping mitigating human PSM.^62^

In the present study, fewer than half of treated dogs showed improvement, and complete resolution was observed in only 2 cases. On the other hand, other dogs received these same medications and did not show improvement. In 1 case, the dog was euthanized due to the high frequency of the episodes (multiple clusters daily) and lack of response to levetiracetam. The owner was elderly and declined additional treatment options and alternative management strategies, including rehoming. The owner perceived the events as extremely distressing and ultimately unmanageable and sought advice elsewhere. At the owner's request, the dog was euthanized. Although this condition appears to have a benign natural course and is not considered by the authors to be associated with relevant suffering, studies in dogs with other types of seizures, like epileptic, indicate that high seizure frequency can substantially impact owner quality of life.^72^^,^^73^ Levetiracetam is an antiseizure medication often used to treat cortical myoclonus (eg, myoclonic epilepsy)^35^ and is believed to exert anti-myoclonic effect by binding to the synaptic vesicle protein 2A and modulating calcium channels.^13^ The origin of PSM, presumably generated in the spinal cord,^6^ could possibly explain why levetiracetam was ineffective in many of the cases. Two dogs received zonisamide (5-10 mg/kg PO q12h) without any clinical improvement. Half of the dogs in this study did not receive any treatment and episodes resolved spontaneously or decreased in frequency in many of them. This included the dog with suspected liver disease, for which no treatment was initiated. Despite receiving no treatment for either condition, the frequency of episodes decreased over time, and during a 67-month follow-up period, the dog did not develop any additional clinical signs consistent with the suspected liver disease. Therefore, the suspected hepatopathy was considered unlikely to be related to the myoclonus in this dog and was interpreted as an incidental finding. It is unclear for the authors if the dogs that received any type of drugs really improved due to the treatment or if the clinical signs just improve when giving time, a phenomenon also reported in other movement disorders, such as paroxysmal dyskinesia.^74^^,^^75^

The main limitations of this study include its retrospective nature, incomplete diagnostic testing, and lack of systematic neurophysiological assessment with measurement of spinal conduction. Polymyography, which is essential for definitive diagnosis of PSM in humans,^6^^,^^38^^,^^57^^,^^60^ was not routinely available. In the absence of polymyography and EEG coupled with EMG recording to rule out premotor potential, the spinal origin of the myoclonus in these dogs cannot be confirmed. Nevertheless, based on phenotypic similarity to the human condition, a nonepileptic movement disorder was diagnosed and the term “PSM” was used for these dogs in this manuscript. Magnetic resonance imaging studies, essential to exclude or confirm spinal cord lesions, were only available in a few dogs and in some of them, only a part of the spinal cord was included. Clinical response to treatment was difficult to evaluate as dogs that were treated received multiple drugs in different periods of time, in different doses and frequencies. There was also little to no information about first day of treatment and first day of clinical improvement in those dogs who did and did not receive treatment.

Based on our study results, it can be concluded that presumptive PSM appears to be a benign spinal movement disorder that affects young dogs and is likely idiopathic in most cases, as no underlying causes were identified. The condition is characterized by extensor myoclonus predominantly affecting the pelvic limbs, with or without trunk involvement, occurring almost exclusively during lateral and sternal recumbency, with variable frequency and duration and preserved mentation. Although some dogs might respond to medical therapy, spontaneous improvement or resolution seems common. Recognition of this phenotype might facilitate accurate diagnosis, appropriate treatment, and informed prognostic counseling.

## Supplementary Material

Supplementary_Material_Phenotypic_aalag211

Video_1_aalag211

Suppl_video_aalag211

## Off-label antimicrobial declaration

Authors declare no off-label use of antimicrobials.

## Institutional animal care and use committee or other approval declaration

Ethical approval was not required for this study because the animal data analyzed were derived solely from a retrospective review of existing medical records. No additional interventions, treatments, or prospective research procedures were performed beyond routine clinical care. Medical record numbers were recorded prospectively for data collection purposes only, and no activities were undertaken that would have necessitated ethical oversight.

## Abbreviations

CSF: cerebrospinal fluid
CT: computed tomography
EMG: electromyography
MRI: magnetic resonance imaging
PSM: propriospinal myoclonus
