# Supplementary material for: Phenotypic characterization of presumptive propriospinal myoclonus in young dogs (2013-2025)
Source: J Vet Intern Med. 2026 Oct 1;40(5):aalag211. doi: 10.1093/jvimsj/aalag211 (PMC13630453; doi:10.1093/jvimsj/aalag211)
Supplement: Supplementary file 2 [file supplementary_material_phenotypic_aalag211.docx]

**Supplementary Material**

**S1**

Details of all MRI machines at different institutions

**AniCura Djursjukhuset Albano**: 0.2T Esaote Vet-MR Grande (Esaote, Genoa, Italy).

**Utrecht University**: 1.5T Philips Ingenia (Philips Healthcare, Best, The Netherlands).

**Maritime Veterinary Emergency and Specialty Centre**: 1.5T Siemens MAGNETOM Sempra (Siemens Healthineers, Erlangen, Germany).

**Veterinary Specialists and Emergency Center**: 1.5T Siemens MAGNETOM Sempra (Siemens Healthineers, Erlangen, Germany).

**CityU Veterinary Medical Centre**: 0.35T GE Ovation (GE HealthCare, Florence, USA).

**Centre Hospitalier Vétérinaire Saint-Martin**: 1.5T Siemens MAGNETOM Sempra (Siemens Healthineers, Erlangen, Germany).
