# Supplementary material for: Phenotypic characterization of presumptive propriospinal myoclonus in young dogs (2013-2025)
Source: J Vet Intern Med. 2026 Oct 1;40(5):aalag211. doi: 10.1093/jvimsj/aalag211 (PMC13630453; doi:10.1093/jvimsj/aalag211)
Supplement: Supplementary file 3 [file suppl_video_aalag211.docx]

**FIGURE/VIDEO LEGENDS**

**Video 1.** Presumptive propriospinal myoclonus phenotype in young dogs.
